# Pediatric Myocarditis: What Have We Learnt So Far?

**DOI:** 10.3390/jcdd9050143

**Published:** 2022-05-03

**Authors:** Elettra Pomiato, Marco Alfonso Perrone, Rosalinda Palmieri, Maria Giulia Gagliardi

**Affiliations:** Department of Pediatric Cardiology and Cardiac Surgery, Bambino Gesù Children’s Hospital, IRCCS, 00165 Rome, Italy; elettra.pomiato@opbg.net (E.P.); marcoalfonso.perrone@opbg.net (M.A.P.); rosalinda.palmieri@opbg.net (R.P.)

**Keywords:** myocarditis, children, endomyocardial biopsy, inflammatory cardiomyopathy, immune system

## Abstract

Myocarditis is an inflammatory disease of the myocardium that is troublesome to diagnose and manage, especially in children. Since the introduction of endomyocardial biopsy (EMB), new diagnostic tools have provided useful data. Especially when enhanced with immunohistochemistry and polymerase chain reaction (PCR) studies, EMB remains the gold standard for the diagnosis. Notably, cardiac magnetic resonance (MRI) is a non-invasive tool that can confirm the diagnosis and has a particular usefulness during the follow-up. The causes of myocarditis are heterogeneous (mostly viral in children). The course and outcome of the illness in the pediatric population represent a complex interaction between etiologic agents and the immune system, which is still not fully understood. The clinical presentation and course of myocarditis vary widely from paucisymptomatic illness to acute heart failure refractory to therapy, arrhythmias, angina-like presentation and sudden cardiac death. In this setting, cardiac biomarkers (i.e., troponins and BNP), although unspecific, can be used to support the diagnosis. Finally, the efficacy of therapeutic strategies is controversial and not confirmed by clinical trials. In this review, we summarized the milestones in diagnosis and provided an overview of the therapeutic options for myocarditis in children.

## 1. Introduction

Myocarditis represents a challenge especially in children, despite being studied since the second half of the 20th century with sensible progress in the diagnostic field over time.

Clinical manifestations of the disease can vary widely and encompass paucisymptomatic illness, acute heart failure sometimes requiring mechanical circulatory support (MCS), arrhythmias, angina-like presentation and sudden death. Biomarkers can be useful to support the diagnosis, but are not specific [1].

The main cause of myocarditis in children is viral, but many other agents can provoke the illness [2].

Moreover, it is well known that the immune system plays a key role in the pathogenesis of the disease [3], but the mechanisms of interaction between etiologic agents and pathogenesis are not completely understood. As a consequence, myocarditis can resolve without sequelae or evolve into inflammatory cardiomyopathy [4], but to date there are no risk factors predicting poor outcome or chronic disease.

Finally, evidence in therapy remains weak and controversial.

In this review, we summarize the milestones in diagnosis and provide an overview of the therapy of myocarditis in children.

## 2. Definition of Myocarditis and Diagnostic Criteria

As stated by the WHO/International Society and Federation of Cardiology (WHO/ISFC), myocarditis is an inflammatory disease of the myocardium diagnosed by endomyocardial biopsy (EMB) using established histological, immunological and immunohistochemical criteria [5].

**Figure 1:**
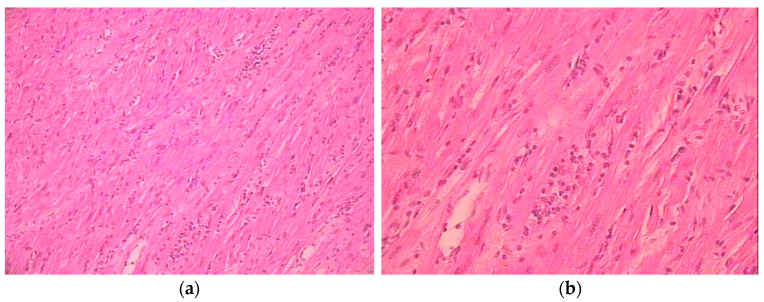
Myocardial histological sample showing lymphocytic infiltrate and myocyte necrosis. (**a**) ×10; (**b**) ×40.

In 1987, Aretz et al. performed the diagnosis of myocarditis on Dallas criteria (Table 1 and Figure 1), on the basis of the presence of inflammatory infiltrate and myocyte necrosis in EMB samples [6]. Even at present, the EMB remains the gold standard for proven myocarditis, despite its invasiveness and the possibility of false-negative results due to sampling errors [2].

In 1991, Gagliardi et al. [7] demonstrated the role of cardiac MRI to confirm myocarditis in pediatric age, and evidence has increased in the subsequent years supporting the importance of the technique also during the follow-up [8]. However, formal criteria (i.e., Lake Louise criteria) were published 18 years later, in 2009 [9] and revised in 2018 [10]. Although many studies were developed in adults, they seem to maintain comparable sensitivity also in children [11], but only single center studies are available in the pediatric population.

Notably, myocarditis can sometimes evolve to inflammatory cardiomyopathy, that is, myocarditis in association with cardiac dysfunction following an autoimmune, infectious or idiopathic pathway [12], eventually ending in dilated cardiomyopathy [1,13].

## 3. Epidemiology

The incidence rate of acute myocarditis in children is reported to be 0.9 per 100,000 children per year in the US, with a rising trend from 2007 to 2016 [14], and up to 1.95 per 100,000 children/year in one national European study [15].

In children, myocarditis has a bimodal distribution with peaks in those younger than 2 years and in teenagers between 13 and 18 years old [16]. Potential risk factors in children with myocarditis include males, Caucasian background, and low socio-economic status. [14]

In-hospital mortality significantly declined over time, being recorded at 6.1% ± 1.3% in 2016 [14].

## 4. Causes

Myocarditis can be caused by several different agents. The most prevalent etiology is infectious (i.e., viral), but many other non-infectious agents can be causative [2].

### 4.1. Viral Myocarditis

Viruses are the most relevant cause of myocarditis in children. The first report of viral isolation in humans was written in 1969 when a Coxsackie virus group B was isolated in the heart of a 15-year-old boy with myocarditis during necroscopy [17], but other viral causative agents were subsequently reported.

Historically, the most frequently encountered agents were Enterovirus (namely Coxsackieviruses B) and adenovirus [18], but Parvovirus B19 [19] and human herpes virus 6 are also emerging [18].

Myocarditis secondary to cytomegalovirus can be life-threating in immunosuppressed patients [20].

In the current pandemic era, SARS-CoV-2 virus is emerging as a possible cause of myocardial injury, and accumulating evidence suggests that in children it can cause a multisystemic inflammatory syndrome with cardiac involvement, especially in patients with congenital heart diseases [21].

Certain recent studies advise caution in addressing viral etiopathogenesis to viral causes due to potential survival of these viruses in different tissues even weeks or months after initial contact [22]. Further studies are needed to derive conclusions.

### 4.2. Infectious Non-Viral Causes

*Trypanosoma cruzi* can cause myocarditis in the form of Chagas disease with major distribution in South America, but increasingly described in different parts of the world. More rarely, bacteria such as *M. tuberculosis*, *Mycoplasma* spp. and *Borrelia* spp., parasitic infestations and *T. gondii* can induce the illness [20].

### 4.3. Noninfectious Etiology

In children, noninfectious agents causing myocarditis are rare events. Autoimmune myocarditis is suspected when EMB shows inflammatory response but fails to detect infective agents and other causes are unlikely. The heart can be primarily involved or affected in the context of systemic autoimmune diseases as rheumatic fever or systemic lupus erythematosus [13,23]. More rarely, other causative agents encompass hypersensitivity reactions, medications (sulfa-drugs and anthracyclines) and toxins [2,20].

Recently, myocarditis after mRNA vaccine against SARS-CoV2 has been reported in adolescents [24]. According to a recent US Survey Study in children from 12 to 18 years old, vaccination-related adverse events occurred in 1/1000 recipients, and myocarditis represented 4.3% of the reported events (0.0043% of all adverse events). Notably, most cases were mild or moderate and resolved favorably [25], as previously reported also in adults [26].

## 5. Pathogenesis

The immune response plays a pivotal role in the genesis and perpetration of myocardial damage, as demonstrated in animal studies [27,28]. However, the interaction of specific viruses (or other causative agents) with the immune system and the role of autoimmunity in the pathogenesis of myocarditis and inflammatory cardiomyopathy are not fully understood [4].

Myocarditis represents the acute phase of the myocardial inflammation and can be viral, post-infectious, immune-mediated or primarily an organ-specific autoimmune condition [13].

### 5.1. Viral Myocarditis

Focusing on viral myocarditis, the pathogenesis of myocardial damage can vary depending on the pathogen. In virus-induced active myocarditis, cardiotropic and vasculotropic viruses, such as adenovirus or enterovirus, directly injure cardiac myocytes or vessels, leading to inflammatory reactions. In virus-associated myocarditis, the pathogen can be detected in tissue samples, but its role in the myocardial damage is not clear as the infection could be latent, such as in herpesviruses and Parvovirus B19. Other viruses can provoke a cytokine storm or activate the cellular immune response through molecular mimicry [4].

Once the virus infects the heart, the immune system initially responds according to the innate pathway (acute phase, day 1 to day 7). The innate immune system encompasses mast cells, natural killer (NK) cells, dendritic cells, neutrophils, eosinophils, basophils and monocytes, but their activation is influenced by the type of pathogen and the molecules expressed by the damaged cardiac myocytes. In particular, monocytes infiltrate myocardium and differentiate into macrophages that produce pro-inflammatory cytokines and activate the adaptive immune response. The innate immune response is, therefore, crucial to overcome viral infection, but it can also cause excessive myocardial disruption and dysfunction, especially when exaggerated or prolonged [29,30].

The switch to the adaptive mechanisms of immunity marks the beginning of the subacute phase that usually resolves the infection within 4 weeks (often in 14 days) and in which the major effectors are the T cells [4]. However, if these mechanisms fail to eliminate the pathogen or if the immune response persists, inflammation becomes chronic and can induce the production of cardiac autoantibodies and autoimmune reaction [31,32,33,34].

In this setting, genetic and external factors interact differently in the pathogenesis of myocarditis [35,36], leading to different clinical pictures and outcomes.

### 5.2. Autoimmune Pathway

In some cases, the immune response is inappropriately raised compared to the need of the organism (hypersensitivity) or is triggered by the loss of self-tolerance (autoimmune post-injury reactions) [33]; in others, myocytes may expose self antigens or neoantigens that can be attacked by autoreactive antibodies. In summary, autoimmune inflammatory cardiomyopathy can be primarily autoimmune or represent the final stage of a pathogenetic pathway in which the causative agents (mainly viruses) interact differently in genetically predisposed patients [13].

As in other autoimmune diseases, at least two of the Rose–Witebsky criteria should be satisfied to diagnose autoimmune illnesses [37]. Referring to myocarditis, it is well known that the inflammatory infiltrate along with an abnormal HLA expression in the myocardium can be detected, without proving the presence of infectious agents or known inflammatory causes [38,39]. Circulating cardiac autoantibodies can be detected in up to 60% of patients with inflammatory cardiomyopathy and in their relatives also many years before the clinical picture becomes manifest [40,41]. Moreover, autoantibody and/or autoreactive lymphocytes have also been detected in myocardial tissue samples of affected individuals [3]. Many classes of autoantibodies have been discovered; they are directed against several autoantigens, mainly α- and β-myosin heavy chain, and some of them can directly damage the myocardium [42,43]. On the other hand, the presence of autoantibodies is important to guide the subsequent immune-modulating treatment [44]. The efficacy of immunosuppressive therapy is still controversial, but recent evidence is encouraging [45,46] (see also Therapy).

## 6. Signs and Symptoms

Another challenging point in the management of pediatric myocarditis is the clinical presentation. Even if the majority of patients complain of symptoms, these can vary widely, ranging from unapparent infections or unspecific symptoms to acute heart failure refractory to therapy, arrhythmias, angina-like presentation and sudden cardiac death [16,47,48].

Fever at presentation can be present in 58% of patients with biopsy-confirmed myocarditis (versus 15% of patients with dilated cardiomyopathy, *p* = 0.002) [49].

In a recently published multicentric cohort study, chest pain and respiratory distress appeared to be more frequent in patients with mildly depressed to normal ventricular function, while gastrointestinal and unspecific symptoms were prevalent in patients with moderately to severely depressed ventricular function. Dyspnea and viral prodromes were equally prevalent in the two groups [16].

The median length of stay in stable hospitalized patients has been reported as 6.1 days. In a recently published German prospective multicentric registry on pediatric myocarditis, factors associated with major cardiovascular adverse events were fulminant presentation, monocytes as inflammatory infiltrate, persistence inflammation and younger age [46].

### 6.1. Heart Failure

According to a recent retrospective, serial cross-sectional study, hospitalization for heart failure related to pediatric myocarditis has remained stable over time (27%) [14].

In one study, the incidence of adverse events including death and heart transplantation was 13%. Risk factors at logistic regression analysis included younger age, female sex and higher left ventricular end-diastolic diameter (LVEDd) z-score at time of presentation [16], but data on predictors of negative outcome are not uniform [50].

Fulminant myocarditis is the clinical manifestation of a rapidly evolving heart failure, as a result of widespread inflammation of the myocardium [51]. In a single-center retrospective study investigating children with fulminant myocarditis, risk factors for cardiac arrest or MCS seemed to be higher peak BNP levels and inotropic scores [52].

### 6.2. Arrhythmias

Arrhythmias are frequent in children with myocarditis. Among rhythm disturbances, tachyarrhythmias are more than twice as common as bradyarrhythmias (13% versus 6.4%) [14]. Ventricular tachycardia is the most frequently encountered rhythm disturbance in hospitalized patients [14], but ventricular tachycardia, ventricular fibrillation, supraventricular tachycardias, and atrial fibrillation or flutter have also been reported [53].

In contrast, complete heart block is the most common bradyarrhythmia in children [54], and its detection with ECG should raise suspicion of myocarditis [14].

### 6.3. Chest Pain and Angina-Like Presentation

Chest pain is rarely due to cardiac pathology in children; nonetheless, it is a well described symptom during myocarditis and pericarditis [55]. It can be associated with ECG changes, an increase in troponin level and abnormalities of regional or global wall motion, mimicking myocardial infarction [56].

### 6.4. Sudden Death

According to data in the literature [57], myocarditis accounts for 8% of the sudden deaths of known cardiovascular cause in young competitive athletes (mean age 19 ± 6 years). In ~75% of cases, the affected individuals were males, and ~50% had one feature among the following: viral prodrome, syncope, unspecific symptoms, chest pain or palpitation [58].

## 7. Diagnosis in the Clinical Setting

**Figure 2:**
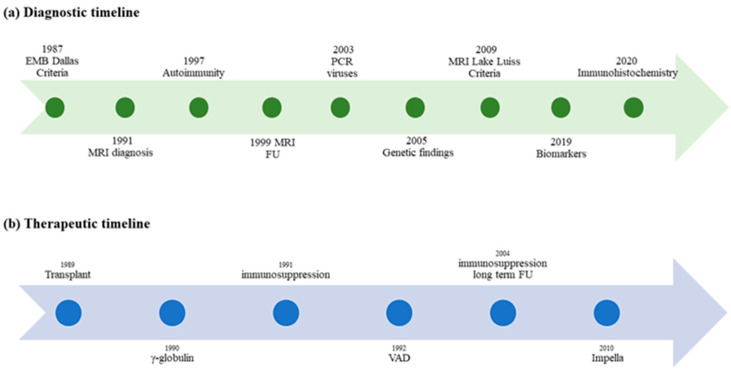
Timelines of milestones in diagnosis and therapy of myocarditis in children. (**a**) Milestones of diagnosis in pediatric myocarditis over time; (**b**) Evidence of therapy in children with myocarditis: the therapeutic approach is not supported by specific pediatric clinical trials.

In the absence of EMB or CMR, the diagnosis of myocarditis in the clinical setting can only be suspected by the merger of different data including medical history, signs, symptoms, biomarkers, electrocardiogram (ECG) and echocardiographic features, as none of these is specific or pathognomonic for myocarditis. Major milestones in the diagnosis of myocarditis in children are shown in Figure 2, Panel a.

### 7.1. Electrocardiography

All patients with suspected myocarditis should be investigated with a standard electrocardiogram (ECG). As almost any other first-line investigation in the field, an ECG is neither sensitive nor specific for the diagnosis [1]; however, it can be useful to add suspicion together with a reasonable clinical presentation, keeping in mind that coronary ischemic disease is very rare in the pediatric population.

In children, an ECG can detect sinus tachycardia, nonspecific repolarization abnormalities, diffuse concave ST-segment elevation, low-voltage QRS complexes in the limb leads [59] and arrhythmias [53].

### 7.2. Echocardiography

Echocardiography is another first-line investigation that should be performed on the suspicion of myocarditis. As with electrocardiography, it lacks sensitivity and specificity, but it can be useful to exclude primary valvular or congenital heart diseases and to monitor the patient over time, thanks to its availability, the possibility of real-time assessment and significant tolerability even in children [1,2].

Among others, echocardiography can detect variable degrees of left or right systolic impairment or left ventricle dilatation, regional wall motion abnormalities, thickened myocardium typically disproportioned compared to ventricular enlargement due to wall edema, pericardial effusion, intracardiac thrombosis, and secondary valvular regurgitation [60].

More recently, tissue Doppler for evaluation of diastolic function [61] and myocardial strain [62] has shown correlation with EBM o CMR findings and ultimately with outcomes [63].

### 7.3. Laboratory Findings and Biomarkers

Common inflammatory biomarkers include erythrocyte sedimentation rate and PCR. They can be elevated in myocarditis as in other inflammatory illnesses, including pericarditis [1].

Traditional cardiac biomarkers encompass troponins, B-type natriuretic peptide (BNP) and N-terminal pro-BNP (NT-proBNP). Troponin levels rise in cases of myocardial injury and can be elevated in acute myocarditis [64]. BNP and NT-proBNP increase in cases of myocardial dysfunction, can discriminate cardiac from non-cardiac causes of dyspnea [65], and peak BNP seem to be a risk factor for poor outcome in children with fulminant myocarditis [52].

Although all of the above-mentioned molecules are unspecific for the diagnosis, they can be useful to support or suspect possible myocarditis, especially in the acute setting. In contrast to the adult population, in fact, other causes of myocardial injury and dysfunction such as coronary diseases are very rare in children. Importantly, these biomarkers are of more value when normal biomarkers do not rule out myocarditis [1].

### 7.4. Endomyocardial Biopsy (EMB)

EMB represents the gold standard for the diagnosis of proven myocarditis following the Dallas criteria listed in Table 1 [6]. The development of immunohistochemistry allowed physicians to differentiate the type of inflammatory infiltrate according to their clusters of differentiation (CDs) and to detect HLA expression. The distinction of leukocytes (CD45+), T lymphocytes (CD3+) and their subtypes (CD4+ or CD8+) and macrophages (CD68+) led to higher detection rates in the diagnosis of myocarditis, improving the sensitivity and specificity of the EMB [66]. According to the Working Group on Myocardial and Pericardial Diseases of the European Society of Cardiology, an inflammatory infiltrate should be defined if ≥4 leucocytes/mm^2^ are detected in the tissue sample. Up to 4 monocytes/mm^2^ can be found in the specimen along with ≥7 T lymphocytes (CD3+)/mm^2^ [1].

Furthermore, IHC examination can also detect the presence of autoantibodies within the damaged myocardium [42,43].

Notably, once the myocarditis is proven or suspected, the viral origin can be confirmed by PCR in myocardial tissue. In 1987, a hybridization in situ technique was used to identify enteroviral RNA in myocardial cells, using molecularly cloned coxsackievirus B3 cDNA as a diagnostic probe [67]. Since then, the PCR technique has been widely used to detect viral genome in EMB samples [18]. This technique can diagnose the specific pathogen, improving the value of the EMB [68]. Contrarily, viral serology of peripheral blood and PCR of peripheral samples (i.e., urine, blood or stool) poorly correlate with the final diagnosis [1]. Good concordance between myocardial and blood PCRs can be a reasonable element in contributing to diagnostic criteria.

Importantly, results from EMB showing negative viral PCR in association with positivity of cardiac autoantibodies indicate an immune-mediated illness and are the basis for a safe immunosuppressive therapy [13].

The two major concerns regarding EMB are the invasiveness and the rate of false-negative results. The complication rate can vary and is higher in children with suspected cardiomyopathy than in heart transplant recipients already on inotropic support [69,70]. However, it is generally a feasible procedure even in very small infants, and in large multicenter studies, the incidence rates of major adverse events related to myocardial biopsy in children with suspected cardiomyopathy in three recent cohorts were 13.2% [71], 5% [70] and 2.6% [46], respectively.

#### Giant Cell Myocarditis (GCM)

EMB plays a major role in cases of rapidly progressive heart failure refractory to medical treatment [1]. The paradigm of such presentation is represented by giant cell myocarditis, also known as Fiedler myocarditis, a distinct and rare type of myocardial inflammation whose first clinical manifestation is frequently cardiogenic shock. Clustered macrophages (i.e., giant cells) and lymphocytes with subsequent heart muscle cell destruction are the histological basis for the myocardial infiltrate.

The first, and for many years unique, case of pediatric giant cell myocarditis was reported in 1955 by Goldberg, who described the case of an infant that died at 6 weeks after birth [72]. GCM is in fact rare in children, and it usually affects young and middle-aged adults and presents rapidly with progressive heart failure with or without electrical instability [73].

The etiology is still not completely understood, but it seems to be mediated by T lymphocyte dysregulation. Its association with multisystemic conditions including autoimmune diseases (inflammatory bowel disease, rheumatoid arthritis, systemic lupus erythematosus, autoimmune thyroiditis, etc.) in 20% of cases or malignant thymoma and lymphoma supports the hypothesis related to T lymphocyte involvement [74,75,76].

The differential diagnosis includes other types of fulminant myocarditis such as lymphocytic myocarditis, eosinophilic myocarditis and granulomatous diseases. Among these, lymphocytic myocarditis is prevalent, while eosinophilic or giant cell fulminant myocarditis are rarer but have to be considered in the differential diagnosis, and EMB is the tool for the right diagnosis [77].

In GCM, an early and appropriate diagnosis is crucial [76] due to poorer prognosis in the absence of combined immunosuppressive therapy and / or MCS (on the basis of clinical presentation) [73]. Prompt MCS is fundamental in fulminant cases to improve survival, preserving multiorgan function as a bridge to recovery or heart transplantation [78].

### 7.5. Cardiovascular Magnetic Resonance (CMR)

The importance of CMR for the diagnosis and follow-up of myocarditis in children has been noted since the early 1990s [7,8]. However, the diagnostic criteria are not specific for the pediatric population and are derived from adult cohorts.

According to the original Lake Louise Criteria published in 2009 [9], the diagnosis of myocarditis is probable if at least two of the following three criteria are satisfied:

(1) Regional high T2 intensity signal or high T2 intensity signal ratio suggestive for myocardial edema;
(2) Increased early gadolinium enhancement (EGE) ratio, suggestive for hyperemia and/or capillary leak;
(3) Presence of late gadolinium enhancement (LGE) of non-ischemic pattern, a sign of non-ischemic necrosis.

The accuracy, sensitivity and specificity of these criteria were 78%, 67% and 91%, respectively [79].

Unfortunately, after the acute phase, the intensity signals of both T2 and EGE become more homogeneous, making subacute inflammation (more) difficult to detect.

In the subsequent years, myocardial mapping of T1, T2, and extracellular volume has emerged as a tool for tissue characterization, since the relaxation time is more significant than the signal intensity [80].

**Figure 3:**
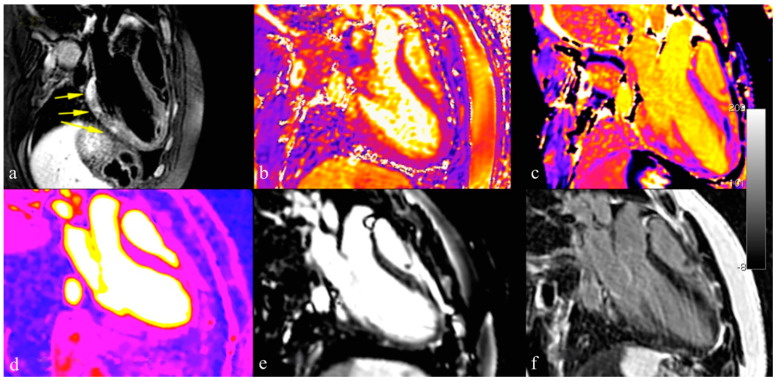
Different techniques for myocarditis assessment with cardiovascular magnetic resonance imaging: T2 STIR sequences reveal areas of myocardial edema in acute setting (panel **a**); T1 and T2 mapping techniques improved detection of myocardial edema and inflammation (panel **b**,**c**); perfusion imaging and early enhancement can be used to detect hyperemia condition (panel **d**,**e**); late gadolinium enhancement sequences confirmed acute myocardial inflammatory damage and assessment of myocardial fibrosis (panel **f**).

Following these technical enhancements, the Lake Louise Criteria were revised in 2018 [10]. Typical features of myocarditis at CMR are shown in Figure 3.

Myocardial inflammation leads to an increase in T1, T2 relaxation time and extracellular volume (ECV). T2 mapping can differentiate acute inflammation from other forms of inflammation; native T1 mapping is less specific for active myocarditis and should be used when negative, to rule out myocarditis. ECV is a marker of fibrosis and inflammation. Importantly, it can also show changes that are undetectable with LGE sequences, pointing out diffuse myocardial inflammation in patients with negative LGE [81].

In a recent meta-analysis, Pan et al. [82] suggest that adding the new parameters to the standard Lake Louise Criteria can be significantly useful in improving sensitivity in diagnosis and management of acute myocarditis [83].

Even if the original and the revised Lake Louise Criteria have been validated only in the adult population, they seem to maintain their value also in the pediatric population [46]. However specific issues including sedation and younger age are related to a lower image quality [84].

## 8. Therapy

Giving the gap in understanding entirely the mechanisms of pathogenesis of myocardial damage, it is of no surprise that data on therapy are inconclusive. Treatment in children recalls that of adults, taking into account that viral pathogenesis and an acute clinical picture are more frequent in the pediatric population compared to adults. Milestones of therapy in myocarditis are shown in Figure 2b.

### 8.1. Symptoms-Based Therapy

Symptoms-based therapy should be the aim in handling pediatric myocarditis in the acute phase. Arrythmias can occur and should be resolved as soon as possible.

According to a recent retrospective, serial cross-sectional study, hospitalization for heart failure related to myocarditis has remained stable at 27% over time [14].

In the post-acute phase, oral HF treatment should be started in children with reduced ejection fraction. Contrarily, the benefit of such therapy in patients with preserved ejection fraction is under investigation [2]. Butts et al. reported 55% of children with heart failure therapy after hospital discharge [16].

### 8.2. Circulatory Support

In case of cardiogenic shock and low cardiac output, the cardiac pump has to be supported by inotropic therapy (milrinone over epinephrine or dopamine). In refractory cases, pediatric MCS can be considered lifesaving [85,86,87].

Apart from ECMO, advanced strategies for selected children affected by myocarditis could include heart transplantation or a durable left ventricle assist device (LVAD). ECMO support is necessary in 6.7% ± 0.9% and VAD in 1.4% ± 0.3% of the children [14]. Importantly, weaning from MCS is observed in a higher percent of myocarditis pediatric patients [46] compared to those affected by dilated cardiomyopathy and congenital heart diseases [88]. However, there are not enough data to derived solid conclusions [89].

In the subset of children with myocarditis, LVAD can be implanted as a bridge to recovery or heart transplantation. The development of new-generation devices and improvements in the management of major complications (bleeding, infection, cerebrovascular events and right ventricular failure) have allowed better outcomes in children with advanced heart failure.

After MCS application, further management can be divided into different settings. Most cases recover with or without sequelae [12,90]. In the absence of recovery, children must be referred for heart transplantation whenever eligible for the procedure [91].

### 8.3. Immunosuppressive Therapy (IT)

Immunosuppressive therapy encompasses corticosteroids alone or in combination with corticosteroid-sparing agents.

Corticosteroids have been studied in myocarditis of rheumatic origin since 1950, but the results of trials in adults were inconclusive [92,93]. In children, only limited studies with prednisone in combination with azathioprine or cyclosporine [45,94] or case series [95] are available.

However, results seem to highlight the efficacy of IT and are substantiated by an encouraging recent metanalysis [96].

In conclusion, the results of trials in adults were inconclusive, but subsequent investigations supported the effectiveness of corticosteroids in the autoimmune phase of the disease if no active viral infection is present [2,3].

### 8.4. Intravenous Immunoglobulin (IVIG)

The use of IVIG has been reported in the treatment of pediatric myocarditis since 1990 [97], even if its pharmacodynamics are poorly understood. IVIG has anti-inflammatory, anti-viral, and immunomodulatory properties [98].

In the study by Drucker and colleagues, IVIG administration in children with myocarditis was associated with an improved recovery of left ventricular function and with a tendency to better survival during the first year after presentation [97], but again, the results are not uniform [99].

### 8.5. Antivirals

Antiviral medications have been used since the 1980s [27,100,101]. A recent statement of the AHA on pediatric myocarditis advises the use of antiviral agents for myocarditis in case of active infection (even with no need for documentation of viral persistence within the myocardium) [2].

## 9. Follow-Up

After the acute phase, pediatric myocarditis can resolve without sequelae in a reasonable percentage of patients. A long follow-up study carried out on children with biopsy-proven myocarditis reported 83% of survival after a follow-up of 13 years [45]. According to a recent prospective study on children with biopsy-proven myocarditis, with a median follow-up time of 11 years, mortality was comparable in acute, chronic and healed myocarditis, ranging from 6% to 9% [46].

During the follow-up period, clinical assessment, ECG and echocardiography should be performed regularly [102,103,104].

At long-term follow up, CMR can be a useful tool to detect fibrosis and persistence of myocarditis despite a normal EF on transthoracic echocardiogram [105,106,107,108].

The role of EMB during follow-up is controversial. However, it might be considered in selected cases when LV dysfunction and increased serum inflammatory biomarkers or PCR are persistent [2].

## 10. Conclusions

Myocarditis is a tricky disease to diagnose and manage. EMB remains the gold standard for proven myocarditis, whose etiology can be further investigated through immunohistochemistry and PCR on myocardial samples.

Nowadays, MRI has an important role in the diagnostic flow-chart and during follow-up also in children.

The etiology is heterogeneous (mostly viral in children), the clinical manifestations are broad-ranging, and knowledge of the pathogenetic mechanism(s) for single patients is needed to tailor the therapy and improve the outcome of these patients.

## Figures and Tables

**Table 1:** The Dallas Criteria.

Diagnosis	Histological Findings
Myocarditis	Presence of inflammatory infiltrate of the myocardium AND necrosis and/or degeneration of adjacent myocytes of non-ischemic pattern (BOTH are requirements for the diagnosis)
Borderline Myocarditis	Too scarce inflammatory infiltrate OR presence of inflammatory infiltrate of the myocardium WITHOUT NECROSIS of the myocytes.
No Myocarditis	Absence of the above-mentioned histological features
